# Adapting the Eliminating Medications Through Patient Ownership of End Results Protocol to Promote Benzodiazepine Cessation Among US Military Veterans: Focus Group Study With US Military Veterans and National Veterans Health Administration Leaders

**DOI:** 10.2196/35514

**Published:** 2022-09-19

**Authors:** Michael A Cucciare, Traci H Abraham, Lakiesha Kemp, Penny White, Kathy Marchant, Hildi J Hagedorn, Keith Humphreys

**Affiliations:** 1 Center for Mental Healthcare and Outcomes Research Central Arkansas Veterans Affairs Healthcare System North Little Rock, AR United States; 2 Veterans Affairs South Central Mental Illness Research Education and Clinical Center Central Arkansas Veterans Healthcare System North Little Rock, AR United States; 3 Department of Psychiatry University of Arkansas for Medical Sciences Little Rock, AR United States; 4 Center for Care Delivery and Outcomes Research Minneapolis Veterans Affairs Health Care System Minneapolis, MN United States; 5 Department of Psychiatry University of Minnesota Medical School Minneapolis, MN United States; 6 Center for Innovation to Implementation Veterans Affairs Palo Alto Health Care System Menlo Park, CA United States; 7 Department of Psychiatry Stanford University Menlo Park, CA United States

**Keywords:** self-taper, electronically delivered self-help, long-term benzodiazepine use, US military veterans, mobile phone

## Abstract

**Background:**

Long-term dependence on prescribed benzodiazepines is a public health problem. Eliminating Medications Through Patient Ownership of End Results (EMPOWER) is a promising self-management intervention, delivered directly to patients as a printed booklet, that is effective in promoting benzodiazepine reduction and cessation in older adults. EMPOWER has high potential to benefit large health care systems such as the US Veterans Health Administration (VHA), which cares for many veterans who use benzodiazepines for extended periods.

**Objective:**

We aimed to adapt the original EMPOWER booklet materials for electronic delivery and for use among US military veterans receiving VHA care who were long-term benzodiazepine users.

**Methods:**

We used elements of Analysis, Design, Development, Implementation, and Evaluation, a framework commonly used in the field of instructional design, to guide a qualitative approach to iteratively adapting EMPOWER Electronic Delivery (EMPOWER-ED). We conducted 3 waves of focus groups with the same 2 groups of VHA stakeholders. Stakeholders were VHA-enrolled veterans (n=16) with medical chart evidence of long-term benzodiazepine use and national VHA leaders (n=7) with expertise in setting VHA policy for prescription benzodiazepine use and developing electronically delivered educational tools for veterans. Qualitative data collected from each wave of focus groups were analyzed using template analysis.

**Results:**

Themes that emerged from the initial focus groups included veterans’ anxiety about self-tapering from benzodiazepines and prior negative experiences attempting to self-taper without support. Participants also provided feedback on the protocol’s look and feel, educational content, the tapering protocol, and website functionality; for example, feedback from policy leaders included listing, on the cover page, the most commonly prescribed benzodiazepines to ensure that veterans were aware of medications that qualify for self-taper using the EMPOWER-ED protocol. Both groups of stakeholders identified the importance of having access to supportive resources to help veterans manage sleep and anxiety in the absence of taking benzodiazepines. Both groups also emphasized the importance of ensuring that the self-taper could be personalized and that the taper instructions were clear. The policy leaders emphasized the importance of encouraging veterans to notify their provider of their decision to self-taper to help facilitate provider assistance, if needed, with the taper process and to help prevent medication stockpiling.

**Conclusions:**

EMPOWER-ED is the first direct-to-patient electronically delivered protocol designed to help US military veterans self-taper from long-term benzodiazepine use. We used the Analysis, Design, Development, Implementation, and Evaluation framework to guide the successful adaption of the original EMPOWER booklet for use with this population and for electronic delivery. The next step in this line of research is to evaluate EMPOWER-ED in a randomized controlled trial.

## Introduction

### Background

Long-term dependence on prescribed benzodiazepines is a public health problem in multiple countries, including the United Kingdom and the United States [1-4]. Benzodiazepines can provide short-term (<4 weeks) benefits, but they also convey significant risks that worsen over time [5]. Adverse outcomes of long-term benzodiazepine use can include cognitive decline, falls, motor vehicle accidents, benzodiazepine dependence, and opioid-benzodiazepine overdose [6-8]. Furthermore, although benzodiazepines can be helpful for improving insomnia or severe anxiety in the short term, over the long term, they can make these conditions worse [9]. Therefore, interventions are needed to help people discontinue long-term use of these widely prescribed drugs. This paper describes an iterative, qualitative approach to adapting a direct-to-patient intervention​—​Eliminating Medications Through Patient Ownership of End Results (EMPOWER)​—​for electronic delivery [10,11]. To better promote benzodiazepine cessation specifically among US military veterans, this project also tailored EMPOWER to this population. US military veterans were the focus because they are at high risk for longer-term benzodiazepine use [12] and associated health consequences [13].

Cognitive behavioral therapy delivered by a provider, coupled with medication tapering, is effective for reducing long-term benzodiazepine use. Among adults with generalized anxiety disorder, those assigned to receive 12 weeks of cognitive behavioral therapy with medication tapering were more likely to discontinue benzodiazepine use after the intervention than control participants (75% vs 37%, respectively) [14]. Although cognitive behavioral therapy delivered by a provider can reduce benzodiazepine use [14,15], limits on patient willingness and resources, including access, time, and cost, pose challenges to delivering professionally administered in-person benzodiazepine cessation interventions to large populations.

Brief interventions delivered in the primary care setting can also lead to significant reductions in benzodiazepine use and are arguably more feasible to deliver to large populations of adults than more intensive interventions such as cognitive behavioral therapy; for example, in a randomized controlled trial, adult long-term users of benzodiazepines received either a structured primary care intervention consisting of education on the risks of long-term benzodiazepine use, a self-help leaflet to improve sleep, and a gradual medication taper with primary care provider follow-up or a written tailored dose reduction schedule or standard care [16]. At 12-month follow-up, there were no differences between the 2 structured-intervention conditions (provider follow-up or written taper schedule), with results showing that more patients in the 2 brief structured​-​intervention groups discontinued their benzodiazepines than those assigned to standard care (45% vs 15%, respectively) [16]. The findings highlight the feasibility and effectiveness of using direct-​to-patient interventions to reduce long-​term benzodiazepine use, although, as with psychotherapy, brief interventions depend on trained professionals to deliver them and patients to be available in person to receive them.

Technology can make possible a less costly and more scalable approach: providing an easily accessible, direct-to-patient intervention that educates individuals about the risks of taking benzodiazepines over the long-term encourages reduction or cessation of benzodiazepine use and provides tools to help patients taper on their own or in consultation with a provider. Mounting such an intervention on the internet brings further advantages: interventions that do not require patients to travel and that can be accessed at any time and from virtually any place could be appealing to those who might not have the desire or access to use in-person care in a clinic setting.

EMPOWER is a promising direct-to-patient self-management intervention developed by Canadian researcher Dr Cara Tannenbaum [10,11]. EMPOWER provides information about the potential risks and harms of long-term benzodiazepine use and presents alternative, effective options for reducing insomnia or anxiety and help with self-tapering. In the EMPOWER study, older adults (n=148) with long-term benzodiazepine use were mailed the EMPOWER booklet and, compared with controls (n=155), were 8 times more likely to discontinue their use of benzodiazepines [10]. Impressively, these results were obtained without direct care by a clinical professional. If EMPOWER were to be adapted for electronic delivery, such as being accessible by desktop computer, tablet computer, or mobile phone and found to be effective in this format, its reach could be further expanded.

The US Veterans Health Administration (VHA) has significant potential to adapt scalable, effective self-management interventions to help veterans reduce their long-term benzodiazepine use. The VHA is a government-financed health care system that offers comprehensive care to >9 million individuals with prior service in the US military. At the VHA, 355,298 veterans were prescribed benzodiazepines in the fiscal year 2016, almost two-thirds (63.6%) of whom took them for ≥3 months [12]. The VHA patient population includes many older adults [17] and many who take prescribed opioids [18], which increases the health risks of long-term benzodiazepine use.

### Objectives

In this paper, we describe an iterative, qualitative approach used to adapt the original EMPOWER protocol for electronic delivery (EMPOWER-ED) and for use among veterans receiving care at the VHA with long-term benzodiazepine use. To achieve these 2 objectives, we used elements of Analysis, Design, Development, Implementation, and Evaluation (ADDIE), a framework commonly used in the field of instructional design, to iteratively develop EMPOWER-ED [19]. The ADDIE framework uses an iterative approach to identify instructional needs and objectives and to obtain feedback from key stakeholders on initial drafts of instructional content and functioning website prototypes. Through formative evaluation, the ADDIE framework can also help to determine program usability, acceptability, and potential for effectiveness [19,20].

For this study, we used the analysis, design, development, and implementation elements of the ADDIE framework to guide our iterative, qualitative approach to adapting EMPOWER for use among veterans and for electronic delivery. To achieve this, we conducted 3 waves of focus group discussions with 2 groups of key VHA stakeholders. Stakeholders were VHA-enrolled veterans who were long-term users of benzodiazepines and national VHA leaders with expertise in setting VHA policy for prescription medication use, including for benzodiazepines, and in developing electronically delivered self-management tools for veterans. In this paper, we present an overview of qualitative findings from the focus group discussions, including themes representing stakeholder opinions and experiences of using benzodiazepines and attempting to taper as well as specific recommendations for adapting EMPOWER for use among veterans and for electronic delivery. We also provide example images of EMPOWER-ED to highlight key adaptations and their rationale for this population.

## Methods

### Participants and Recruitment

The principal investigators (PIs; MAC and KH) of this study chose to form 2 focus groups: one comprised US military veterans and the other comprised national VHA policy leaders. These 2 types of stakeholders were chosen because of their relevance to the aim of the study, which was to adapt an intervention protocol for the US veteran population and for electronic delivery. Stakeholders were also chosen because the study PIs (MAC and KH), who are both middle-aged White male psychologists and senior health services researchers at the VHA, had access to these populations of individuals at their respective sites [21].

We recruited veterans (n=16) with at least one primary care visit in the prior year at one of the 2 study sites (Veterans Affairs Palo Alto Health Care System in California and Central Arkansas Veterans Healthcare System in Arkansas) and with electronic medical record evidence of long-term or ≥3 months of continuous prescription benzodiazepine use. The purpose of recruiting veterans was to help ensure that EMPOWER-ED content was acceptable to this patient population and reflected their experiences and care needs. Potentially eligible veterans were identified in the corporate data warehouse, which contains data from the VHA’s electronic health record. Veterans meeting initial study eligibility, such as having ≥1 primary care visit in the last year and ≥3 months of a continuous benzodiazepine prescription, were identified and randomly sent letters inviting them to participate in the study.

We also recruited national VHA leaders (n=7) with expertise in VHA care policy for prescription medication use, including benzodiazepines, and in the development and implementation of a web-delivered direct-to-patient educational intervention. The inclusion of national VHA policy leaders helped to ensure that the content of EMPOWER-ED was consistent with VHA priorities and that information was conveyed using up-to-date instructional design techniques. As there is a limited number of national VHA policy leaders with expertise in the aforementioned areas of interest, we used nonrandom selection, which involved identification by the 2 study PIs (MAC and KH) to invite these individuals to participate. We also attempted to recruit VHA primary care providers at the 2 study sites but were unsuccessful.

Each set of stakeholders was invited to participate in all 3 waves of focus group discussions over a 12-month period lasting on average 60 minutes; 16, 14, and 9 veterans participated in waves 1, 2, and 3, respectively, whereas 6, 6, and 5 national VHA leaders participated in waves 1, 2, and 3, respectively. Each stakeholder type participated in a focus group discussion with participants of the same type. Focus groups with veterans took place between October 2020 and September 2021, whereas focus groups with national VHA policy leaders took place between March 2021 and September 2021.

### Description of the Original EMPOWER (Booklet) Protocol

The original EMPOWER protocol consists of an 8-page booklet that was mailed directly to older adults with long-term benzodiazepine use [10,11]. The booklet includes a self-assessment of the potential risks of long-term benzodiazepine use; evidence of benzodiazepine-related harms; knowledge statements designed to evoke cognitive dissonance about the safety of using benzodiazepines; education about possible drug interactions; a vignette depicting a peer who has successfully stopped using benzodiazepines to support self-efficacy to change medication use; information about equally or more effective therapeutic alternatives for managing sleep difficulties or anxiety; and recommendations and guidance for self-tapering their medication, including a taper schedule. The taper schedule consists of a 21-week protocol with daily guidance, regardless of original dose, for reducing medication use. The taper schedule also includes the recommendation for the patient to discuss their self-taper with their physician or pharmacist.

### Data Collection

Focus groups, conducted through the web, were the primary method of data collection. All focus group discussions were audio recorded to facilitate qualitative data collection and analysis. Veterans and national VHA leaders participated in focus group discussions by teleconference using Microsoft Teams, a web-based meeting portal that allows participants to view the same content simultaneously. All focus group discussions were comoderated by the 2 study PIs (MAC and KH), both of whom are clinically trained and have extensive experience in qualitative interviewing and facilitating focus group discussions. An interview guide was developed for the first wave of focus groups to ensure that the areas discussed remained roughly consistent across stakeholder groups and that all relevant topics were addressed. Constructs from the Consolidated Framework for Implementation Research (CFIR [22]) were used to inform the development of the initial interview guide (Textbox 1). The CFIR was chosen because it provides a framework of constructs that can help to identify strategies for optimizing the adoption of a new innovation. In this study, we used the CFIR constructs of intervention characteristics, which includes perceived effectiveness and relative advantage; outer setting, which includes patient care needs and available resources; and characteristics of individuals, which includes knowledge, attitudes, and perspectives toward change [22], to guide the wave 1 focus group discussions. The interview questions for waves 2 and 3 were shorter, less formal, and focused on obtaining feedback on each subsequent draft of the EMPOWER-ED website. Example questions for waves 2 and 3 included “To what extent were your suggestions implemented in the current draft as you envisioned?” “To what extent are draft materials clear and functioning properly?” In addition, the study’s lead qualitative researcher (THA) recorded observations during each wave of focus group discussions. The observations focused on group dynamics and instances in which participants diverged from the questions asked by the comoderators (MAC and KH), including the content and duration of these interactions.

**Figure 1:**
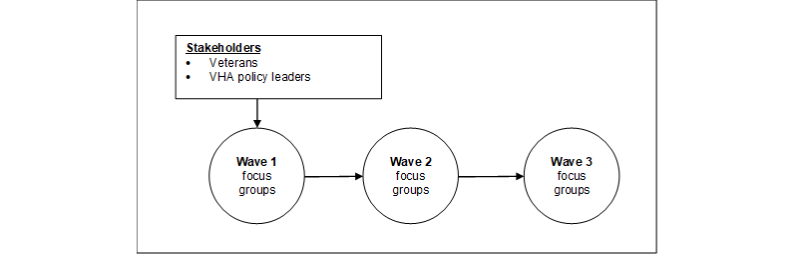
Participant flow through the 3 waves of focus groups. VHA: Veterans Health Administration.

The same stakeholders were invited to participate in all 3 waves of focus group discussions (Figure 1). This facilitated consistency of feedback over time because participants were aware of the recommendations provided during prior waves and could provide feedback on whether recommendations were incorporated as desired into the current draft. Furthermore, for focus group waves 2 and 3, stakeholders were asked to interact with the draft website in the week before the focus group to facilitate discussions on user experience. At the start of the second and third waves of focus groups, the comoderators (MAC and KH) reviewed the intervention materials, described the adaptations made to content, and demonstrated website functionality.

**Interview guide for wave 1 focus groups. After each series of questions, assessing each Consolidated Framework for Implementation Research domain, the focus group facilitators obtained initial recommendations and suggestions for adding, deleting, and modifying elements to the Eliminating Medications Through Patient Ownership of End Results (EMPOWER) materials for adaptation for electronic delivery and to optimally assist veterans in reducing or discontinuing their long-term benzodiazepine use.**

**Wave 1 focus group interview guide**

- Characteristics of EMPOWER
- “What are your thoughts about the content of EMPOWER?”
- “What are your thoughts and opinions about the educational materials? The vignette? The taper schedule?”
- “To what extent would each of these components of EMPOWER be helpful (or not) to veterans in reducing their use of benzodiazepines?”
- “What are your thoughts and opinions about the length of the EMPOWER materials?”
- “What changes (if any) would need to be made to EMPOWER to improve its usefulness for veterans?”
- Outer setting
- “To what extent do you feel that veterans using benzodiazepines would respond (or not) to this intervention?”
- “What would increase their positive response to these materials?”
- Characteristics of individuals
- “What challenges do you see veterans taking benzodiazepines might have self-tapering their medication?”

### Ethics Approval

The study was approved by the VA Central Arkansas Veterans Health Care System Research and Development Committee and Institutional Review Board (protocol #1527634).

### Data Analysis

Rapid analytic techniques informed by Sobo et al [23] and Hamilton and Finlay [24] were used to quickly produce recommendations for adapting EMPOWER for use among veterans and for electronic delivery. The lead qualitative researcher (THA) developed a prototype summary template in a Microsoft Word document with domains related to the goals of the study; for example, the researcher captured stakeholder feedback and recommendations on the protocol’s look and feel, educational content, tapering protocol, and website functionality.

The lead qualitative researcher (THA) also captured emergent themes and created an *other* domain to record observations made during the focus group discussions, including the content and duration of instances in which participants spoke on issues among themselves.

For each wave of focus groups, the lead qualitative researcher (THA) first listened to the audio recording of the discussion and systematically populated the template categories with data. Content analysis was used to ensure that the full range of experiences, perspectives, and feedback was included in the templates [25]. Template data largely consisted of paraphrased content from the discussions, reflecting stakeholder recommendations, reactions, concerns, and questions. Particularly impactful statements were transcribed verbatim into the templates. After this initial step, the lead qualitative researcher (THA) synthesized individual templates from each wave of focus groups by stakeholder type into 1 template. In some waves, multiple focus groups took place for a specific stakeholder type (eg, 2 separate focus group discussions for different veterans) to accommodate their schedules. When this occurred, data were synthesized into 1 template for that stakeholder group for that specific wave of focus groups.

In the final step, the lead qualitative researcher (THA) synthesized the 2 templates by stakeholder group into 1 summary template containing all feedback for that wave. The researcher also carefully reviewed the *other* domain to identify any themes occurring across focus group discussions. Themes identified were mapped to the CFIR domains and included in the summary template. The comoderators (MAC and KH) of the focus groups reviewed the summary template for completeness and accuracy.

### Finalizing Adaptations

After each of the 3 waves of focus groups, the 2 project PIs (MAC and KH) met with the software development team to review the summary template, which included feedback from both sets of stakeholders. Recommendations deemed to be of high priority, such as being consistent with the original EMPOWER materials or suggested by multiple stakeholders, and feasible, such as being within the cost and time parameters needed to implement the recommendation, were identified and implemented. In waves 2 and 3, participants were asked to review the website modifications to determine whether they were implemented as suggested and provide additional feedback or recommendations.

## Results

### Overview

In the following sections, we first present the themes that emerged from the qualitative analysis, which included veterans’ anxiety about self-tapering and their experiences of attempting to self-taper without support (Textbox 2). We then present focus group participants’ feedback and recommendations on modifying the look and feel, educational content, tapering protocol, and functionality of the EMPOWER-ED website to optimize its adaptation for electronic delivery and use among veterans. Although we attempted to recruit primary care physicians to participate in the focus groups, we were unsuccessful. The primary reason reported by physicians for not participating was that primary care clinics were focusing efforts on the ongoing COVID-19 pandemic, and they had limited time to contribute to non–patient-care activities, including research.

**Emergent themes as well as feedback and recommendations on Eliminating Medications Through Patient Ownership of End Results (EMPOWER) and Eliminating Medications Through Patient Ownership of End Results Electronic Delivery (EMPOWER-ED).**

**Themes that emerged from focus group participants’ feedback and recommendations**

- Emergent themes
  - Veterans’ anxiety about self-tapering their benzodiazepines
  - Veterans’ prior negative experiences attempting to taper without effective, alternative help options for managing sleep and anxiety
- Focus group feedback on the EMPOWER and EMPOWER-ED content
  - Look and feel
    - Include images that reflect veterans’ diversity
    - Develop multiple peer vignettes that include a diverse group of veterans
  - Educational content
    - Clarify names (brand and generic) of commonly prescribed benzodiazepines
    - Emphasize potential risks of longer-term benzodiazepine use
    - Include alternative help options, including websites and mobile apps, for managing sleep and anxiety
  - Tapering protocol and website functionality
    - Encourage veterans to inform their prescribing provider of their decision to taper their benzodiazepines
    - Include clear language instruction for veterans on how much medication to take each day during the taper process
    - Allow veterans to save their taper schedule on the website
    - Ensure that the taper schedule is readable on all devices, including smartphones, tablet computers, and desktop computers

### Emergent Themes

Two themes emerged from analysis of the veterans’ interactions during the 3 waves of focus group discussions related to the CFIR domain Characteristics of Individuals. The first theme reflected veterans’ anxiety about self-tapering, specifically concerns about how to manage sleep or anxiety without their medication. Illustrating this anxiety, a veteran stated, “It’s hard to get some of those war-time experiences out of your head...so being without the medication can be scary” (focus group 3, veteran 03).

An additional theme reflected the veterans’ prior negative experiences of attempting to self-taper without effective, alternative help options for managing sleep or mental health symptoms; for example, a veteran described attempting to self-taper in this context as feeling like “...falling into a bottomless pit” (focus group 2, veteran 04). Similarly, the veterans repeatedly expressed a strong perceived need for having effective resources and support during the self-taper process:

I believe that if anyone decides to taper off their medication, they need to have...assistance. When it is your first time being tapered off, you know you want to be counseled.

Focus group 2, veteran 01

### Focus Group Feedback on the EMPOWER and EMPOWER-ED Content

#### Look and Feel

**Figure 2:**
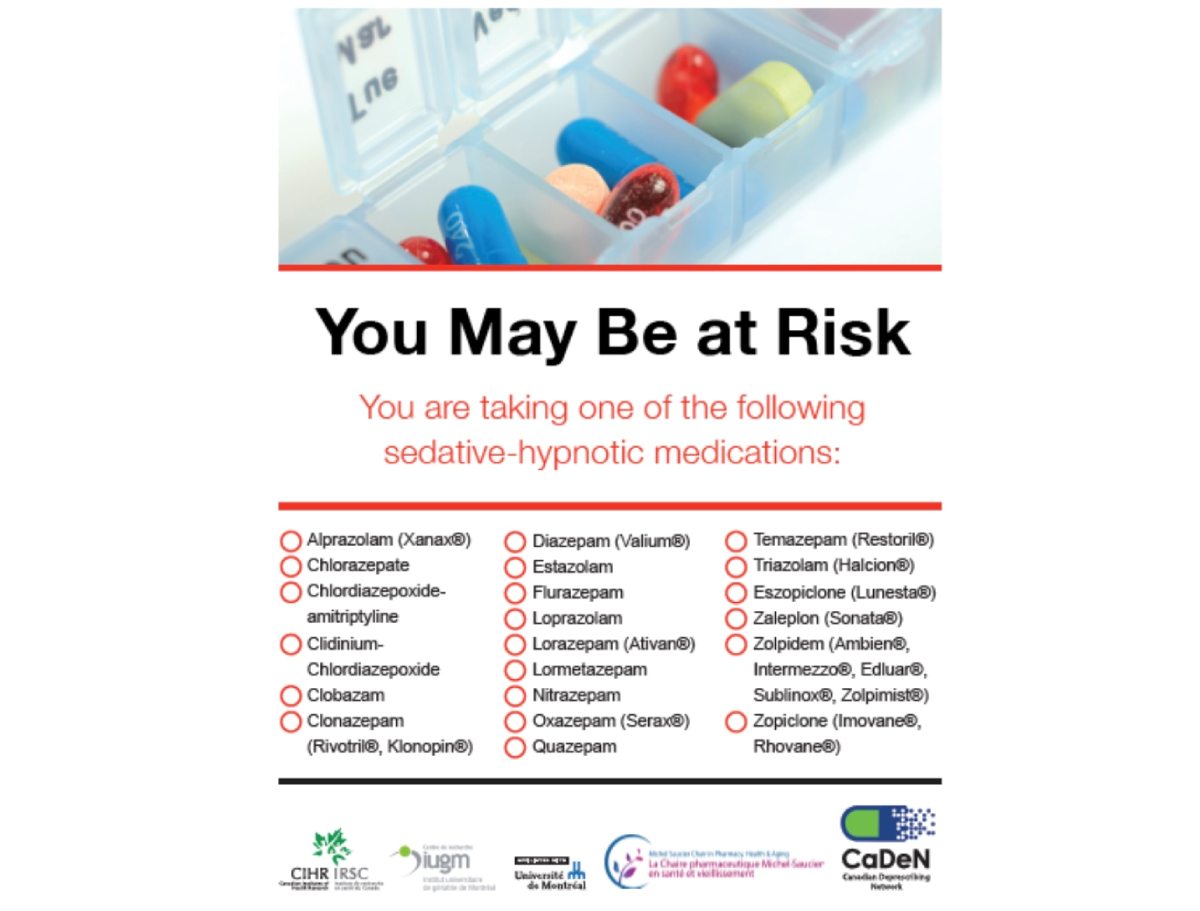
Cover page of the original Eliminating Medications Through Patient Ownership of End Results booklet.

**Figure 3:**
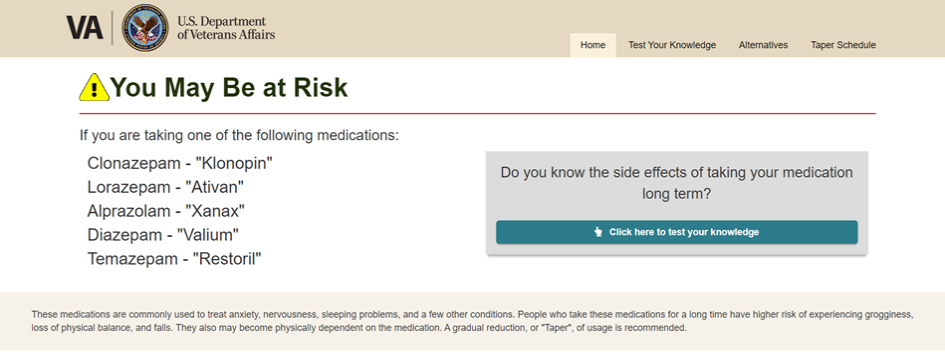
Cover page of the Eliminating Medications Through Patient Ownership of End Results Electronic Delivery website.

Initial focus group feedback centered on modifying the look and feel of the website, including increasing the font size when possible, using less white space, adding more color, and ensuring that the text was at a sixth grade reading level. Participants suggested that we reduce the number of benzodiazepines listed on the website’s home page to those most commonly prescribed at the VHA and use generic and brand names to facilitate identification of these medications by veterans (Figures 2 and 3; modifications to the cover page included simplifying the list of *qualifying* medications that veterans may consider tapering [covering approximately 99% of the benzodiazepine prescriptions at the VHA], including generic and brand names of benzodiazepines to facilitate understanding of which medications are *eligible* for tapering and providing education about the use and risks of these medications over the longer term).

**Figure 4:**
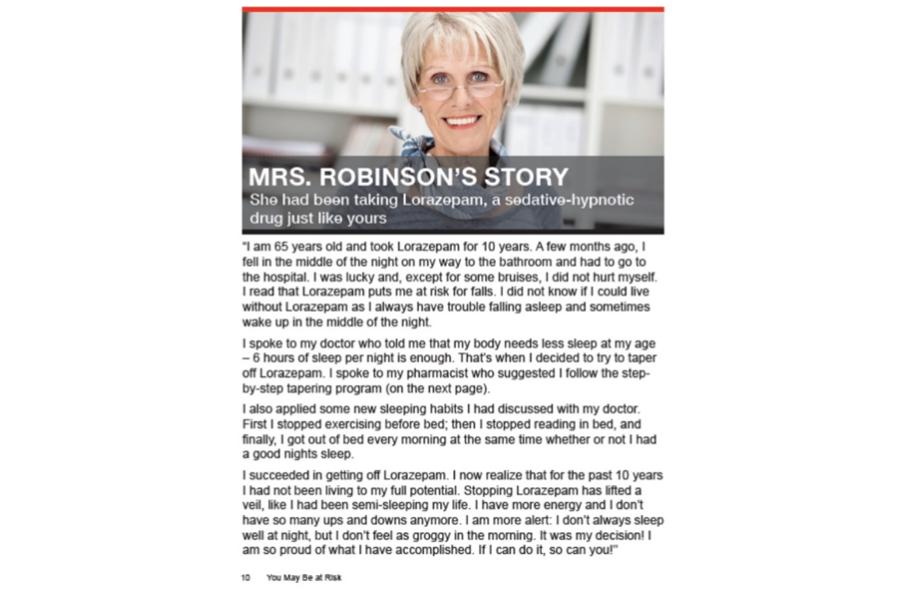
Vignette depicting peers who have successfully discontinued benzodiazepines—the original Eliminating Medications Through Patient Ownership of End Results booklet.

**Figure 5:**
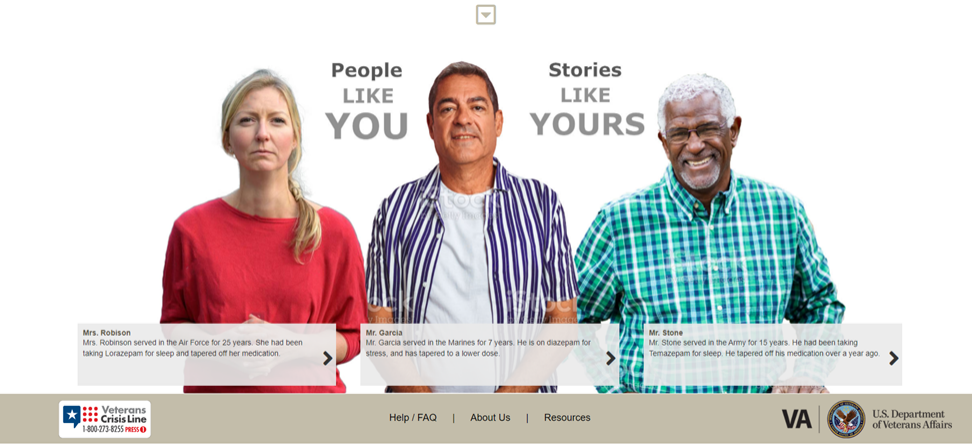
Vignette depicting peers who have successfully discontinued benzodiazepines—the Eliminating Medications Through Patient Ownership of End Results Electronic Delivery website.

Participants recommended adding new images of veterans throughout the program to better reflect their diversity in age, gender, and race; for example, a veteran stated that “...veterans want pictures and stories that are a mix of both ages and races” (focus group 1, veteran 02). Furthermore, a VHA policy leader suggested including “...more realistic photos that don’t look staged and are images of people in a more natural environment” (focus group 2, VHA policy leader 1). This suggestion was most notable for the original peer vignette that depicted an older (aged >65 years) White woman’s story about discontinuing her benzodiazepine use that participants did not generally identify with. Participants recommended that we redesign the single peer vignette content to include 3 diverse peer vignettes to give veterans more choice in selecting a vignette to read or listen to, thus increasing the likelihood that veterans will identify with the content. Consistent with this recommendation, a veteran stated, “...replace reference to Mrs Robinson [woman depicted in the vignette] to different people from different backgrounds to make the materials [vignettes] more relatable to veterans” (focus group 1, veteran 03). Feedback on the peer vignettes also included reducing the length of each peer’s *story*, creating an option to listen to the vignettes through audio as opposed to requiring that they be read and to ensure that veterans from diverse age groups were represented in the vignettes (Figures 4 and 5; modifications to the peer vignettes included developing 3 vignettes [replacing the original single vignette] to provide a more diverse [age and race] representation of veterans. We also provide the option for veterans to listen to each vignette in case of reading or visual impairment [not shown in image]).

In the third wave of focus groups, participants *validated* the final version of the EMPOWER-ED website by indicating that the final images, text, and peer vignettes resonated with their experiences and care needs and reflected the racial, ethnic, and gender diversity of US military veterans.

#### Educational Content

Recommendations regarding the educational content included providing a list of benzodiazepines that are included in the VHA’s National Formulary, using generic terms for medications to educate veterans on which medications could *qualify* for the self-taper, and emphasizing the potential risks associated with longer-term benzodiazepine use and describing those risks, including memory and concentration problems, daytime fatigue, falls, fractures, and motor vehicle accidents, in understandable language. Indeed, a VHA policy leader recommended that we “...organize the list of benzos by most common [commonly prescribed at the VHA] on the first page using large font” (focus group 2, VHA policy leader 3). Regarding how to structure content about the risks of long-term benzodiazepine use, a veteran stated, “...include references to risks that are more common to veterans like drinking in combo with the meds and memory changes and falls” (focus group 2, veteran 05). In addition, participants suggested clarifying how physical dependence on benzodiazepines can occur and to provide content that educates veterans on how an effective taper process, such as a longer taper compared with a shorter one, can minimize withdrawal symptoms.

For alternative help options to medication, participants recommended that we embed links to VHA-developed websites that consist of evidence-based content to improve sleep (Path to Better Sleep) and reduce anxiety (Moving Forward: Overcoming Life’s Challenges). One veteran stated as follows:

...you need additional support during the tapering process. You can get anything you want on the street, so just taking them off is not going to work. I think that just a little help for them [veterans]...would be good. Maybe there could be a support group that is geared toward benzodiazepine cessation.

Focus group 1, veteran 03

**Figure 6:**
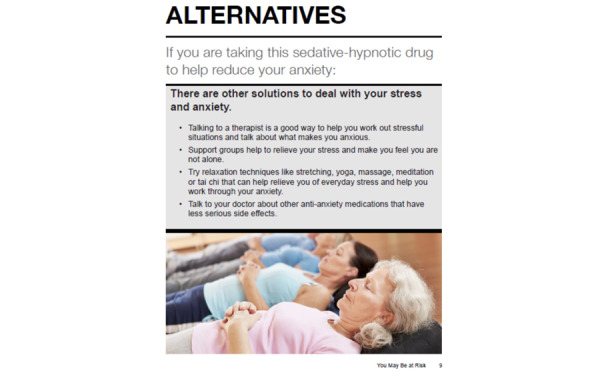
Alternative care and help options for managing anxiety without medications—the original Eliminating Medications Through Patient Ownership of End Results booklet.

**Figure 7:**
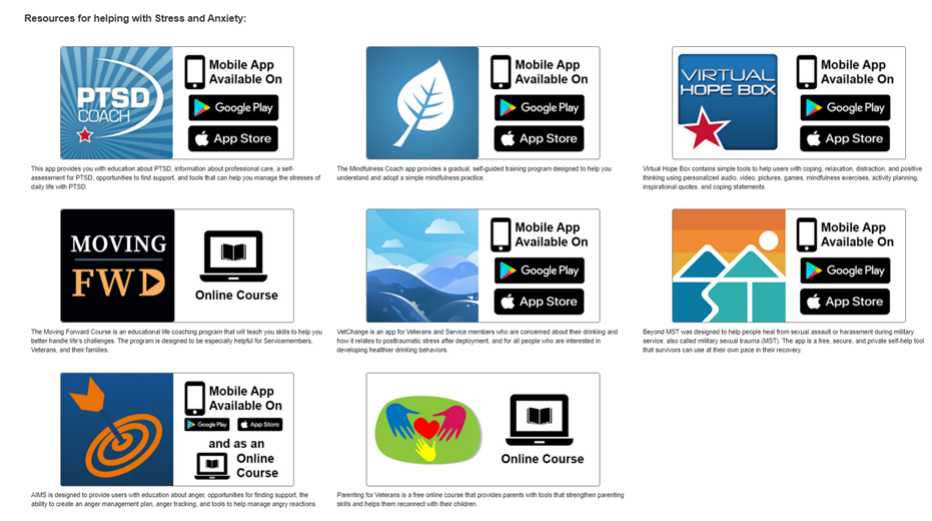
Alternative care and help options for managing anxiety without medications—the Eliminating Medications Through Patient Ownership of End Results Electronic Delivery website.

In addition, participants highly recommended adding “...a link to a website or mobile app that talks about yoga, some kind of meditation, things of that nature” (focus group 2, VHA policy leader 4). As a result, we added VHA help options that can be accessed by mobile phone, such as CBT-i Coach, Insomnia Coach, PTSD Coach, Mindfulness Coach, Beyond MST, VetChange (coping with posttraumatic stress disorder), and Virtual Hope Box (relaxation and stress-coping tools; Figures 6 and 7; modifications to the alternative help and care options section included adding stakeholder [veteran and national VHA policy leader]–suggested website and mobile apps developed by the VHA to help veterans better manage and cope with common mental health comorbidities, eg, posttraumatic stress disorder and anxiety, and other experiences that can cause stress and anxiety as well as learn coping skills for managing stress as well as problem-solving and parenting skills). On the basis of participant feedback, we also clarified which alternative help options are downloadable when using a smartphone, tablet computer, and desktop computer. Furthermore, it was suggested that we add the VHA Crisis Line and links to documents that provide psychoeducation on basic sleep hygiene. In the third wave of focus groups, the veterans *validated* these changes and reported that the modifications to the educational content were clear and enhanced their readability and that the alternative help options included reflected their care needs.

#### Tapering Protocol and Website Functionality

Participants suggested that we emphasize that veterans who choose to self-taper should inform their provider of their decision. A veteran summarized the potential challenge of tapering off benzodiazepines and the importance of a careful, slow taper: “I think it would be a very slow process and then you have to be very careful...you will have to set up a [taper] system where if you are going to get off of them, you need to write a note that reminds you what you need to take [each day]*”* (focus group 1, veteran 03). Furthermore, participants recommended that we emphasize that veterans consider speaking with their prescribing provider or a pharmacist if they need assistance while tapering, provide a link to their local VHA pharmacy to obtain a pill cutter, and more prominently display their daily taper dose provided on the taper schedule.

**Figure 8:**
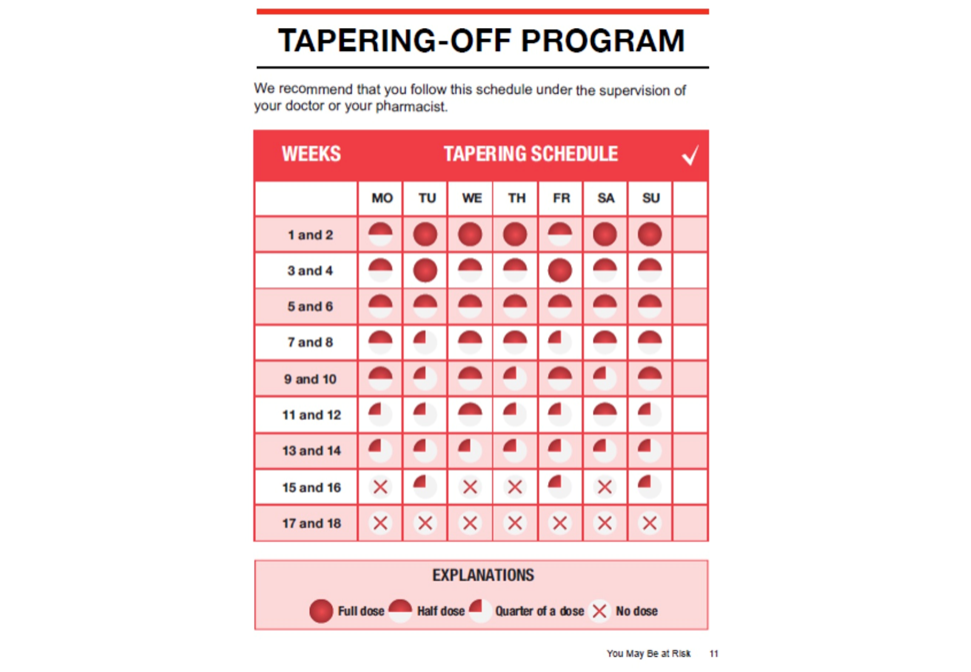
Taper schedule included in the original Eliminating Medications Through Patient Ownership of End Results booklet.

**Figure 9:**
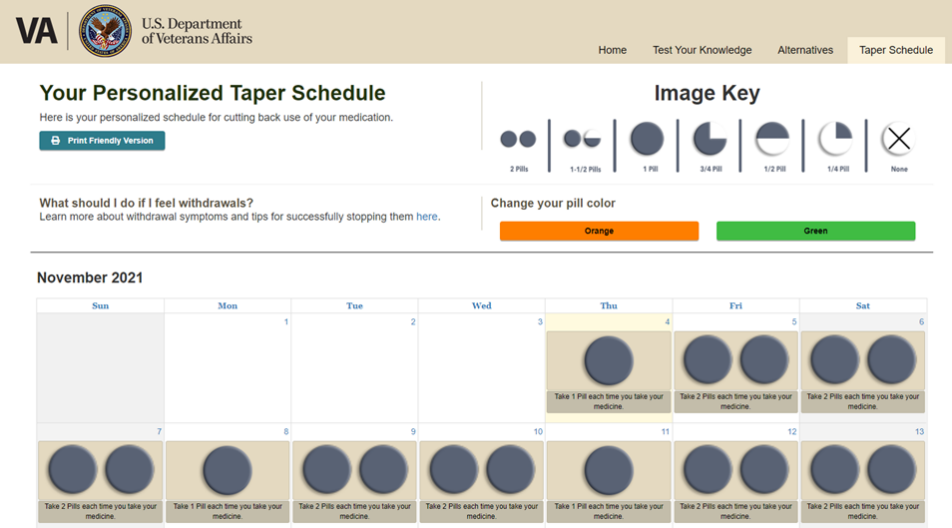
Taper schedule included in the Eliminating Medications Through Patient Ownership of End Results Electronic Delivery website.

Participants also recommended using simpler language such as *reduce* versus *taper* and clarifying that the daily recommended medication dose on the taper schedule is what you take each time you take your medication as opposed to representing the total dosage for that day (Figures 8 and 9; modifications to the taper schedule included making the key depicting the amount of medication to take each day more prominent. We also provide the option for veterans to personalize the taper schedule to accommodate their daily dose and provide written instruction for how much medication to take each day. The program also allows veterans to verify that their daily dose is correct before it generates the personalized taper schedule [not shown in image]). Highlighting this recommendation, a VHA policy leader stated, “...be more explicit about what the person should take, including how many times a day are you taking this dosage and then integrate that into the tapering program” (focus group 2, VHA policy leader 1). Other suggestions included allowing veterans to customize their taper schedule by entering their taper start date and current dosage, including how much they should take each time they take their medication as well as frequency of daily pill taking, implementing an *accuracy check* to ensure that veterans have correctly entered their daily dosage, and allowing veterans to be able to save their taper schedule in the event it needs to be reprinted.

Feedback on website functionality consisted of fixing observed *bugs*, including enhancing the readability of the taper schedule when viewing on a tablet computer or smartphone and allowing users to move freely throughout the website as opposed to requiring that veterans complete a section before advancing to the next.

During the third wave of focus groups, participants *validated* changes made to the taper schedule and reported that the modifications made increased its accuracy, understandability, and potential effectiveness to help veterans taper their benzodiazepines. In addition, participants reported that the modifications made to the website’s functionality reflected their experiences and care needs.

## Discussion

Findings from the 3 waves of focus groups with 2 distinct types of stakeholders revealed emergent themes, including veterans’ concerns about self-tapering their benzodiazepines and prior negative experiences of attempting to self-taper these medications without support. We also received feedback and recommendations on modifying the EMPOWER protocol, including on its look and feel, educational content, tapering protocol, and website functionality, which is described in more detail in the following section.

### Principal Findings and Comparison With Prior Work

Engaging 2 groups of stakeholders in each of 3 waves of focus groups helped us to identify veterans’ concerns about medication tapering and needed adaptations and modifications to the EMPOWER materials to improve their usability and potential effectiveness. Taking into account the needs and preferences of stakeholders or *end users* is critical to designing engaging and effective patient self-management tools [26]; for example, participants from both stakeholder groups emphasized the importance of having access to supportive resources and help options to help manage sleep difficulties or anxiety in the absence of taking benzodiazepines. This resulted in adding several VHA-developed mobile apps and websites with evidence-based content for coping with sleep difficulties and mental health comorbidities, including anxiety and stress. Although EMPOWER-ED is designed to support the self-taper of benzodiazepines, feedback from policy leaders provided valuable insight into the importance of encouraging veterans to discuss their decision to reduce or stop using their medication with their provider. This would facilitate providers being informed of the veterans’ intent and providing assistance when needed, as well as reducing the potential for *stockpiling* medication by reducing automatic medication prescriptions being sent to the veterans’ homes.

As focus group participants were able to interact with the EMPOWER-ED website between focus groups, they also identified potential usability challenges. Feedback from policy leaders during this process directly informed the need to list, on the cover page, the most commonly prescribed benzodiazepines at the VHA so that veterans would be aware of medications that *qualify* for tapering. Both groups of stakeholders emphasized the need to ensure that the taper schedule was clear, including ensuring that veterans’ daily dosage was accurate, before generating their personalized taper schedule. Furthermore, both stakeholder groups suggested strongly that veterans should be able to access their taper schedule in a readable form on a smartphone and tablet computer. This latter feature was important because our intention was to design EMPOWER-ED to be accessible through any electronic device. As a result of these modifications, both groups of stakeholders approved the final version of the EMPOWER-ED website, reporting that the content reflected veterans’ care needs; was accurate, understandable, and easy to navigate; and functioned well. Indeed, providing high-quality educational content and having easy and well-functioning navigation can help facilitate high engagement with patient self-management websites [27].

By including both stakeholder groups, we were able to leverage different perspectives and opinions about EMPOWER-ED to optimize its potential uptake and effectiveness among this population of veterans. We found it particularly beneficial to attend to instances in which veterans went *off topic* to share their experiences of attempting to self-taper from benzodiazepines without support. Allowing veterans to share their prior experiences of attempting—and failing—to self-taper from benzodiazepines underscored the vital importance of additional support during the tapering process, resulting in an end product that more accurately reflected veterans’ preferences and needs. Thus, although recommendations from VHA policy leaders were critical for identifying educational content and optimizing their clarity, the veteran focus groups resulted in adaptations that could ultimately enhance uptake of the intervention among veterans interested in making a change in their long-term use of benzodiazepines. Having both stakeholder groups provide input helped the team to develop a version of EMPOWER-ED that was acceptable and personalized to the needs of the stakeholder groups—all of which are considered key features of self-management websites that promote a positive user experience and clinical outcomes [28].

### Strengths and Limitations

A strength of this study is that it used an instructional design framework (ADDIE) to guide an iterative, qualitative approach to adapting EMPOWER [10], a protocol for promoting benzodiazepine cessation, for veterans with long-term benzodiazepine use and for electronic delivery (EMPOWER-ED). The ADDIE framework embraces an iterative formative evaluation [29] focused on assessing individuals’ care needs, the need for adaptations and modifications, and the usability and usefulness of new e-learning products. The ADDIE framework also emphasizes the importance of obtaining key stakeholder feedback to inform the design and development of educational protocols. Advantages of including stakeholder feedback are having an end product that accurately represents the needs of stakeholders, is acceptable and feasible for use, and is easy to use and navigate [30]—all characteristics that can have a substantial effect on the uptake and effectiveness of e-learning tools [31]. Consequently, the ADDIE framework is increasingly being used in health care to design e-learning products that aim to change end-user behavior; for example, the ADDIE framework has been used to guide the development of e-learning protocols that help people to connect to supportive employment [30], adjust to injury and cope with pain [32], and improve mobility and physical functioning [31].

This study also includes some limitations. First, we did not conduct formal usability testing on the EMPOWER-ED website. As a result, we may have overlooked important issues concerning its usability and functioning with respect to our target population. However, before focus group waves 2 and 3, stakeholders were emailed a link to the most recent iteration of the draft website with the request that they interact with it to facilitate discussion in the subsequent focus group. During the focus groups, it was clear during discussions that some of the participants had interacted with the website, but this was not systematically assessed. Second, despite our attempts, we were not successful in recruiting VHA primary care physicians to give us feedback on the development of EMPOWER-ED. Thus, we may have failed to identify important design or functional modifications that may be important to physicians when helping veterans to self-taper from these medications.

### Conclusions and Next Steps

To our knowledge, EMPOWER-ED is the first direct-to-patient electronically delivered protocol designed to help US military veterans with evidence of long-term benzodiazepine use self-taper their benzodiazepines. In this first step of our research program, our team followed the ADDIE framework to guide the successful adaption of the original EMPOWER booklet [10] for use with this population and for electronic delivery. This resulted in the successful development of EMPOWER-ED. The next step in this line of research is to formally evaluate the effectiveness of EMPOWER-ED in a randomized controlled trial, including examining its impact on veterans’ benzodiazepine use, anxiety symptoms, sleep quality, and overall health.

## Abbreviations

ADDIE: Analysis, Design, Development, Implementation, and Evaluation
CFIR: Consolidated Framework for Implementation Research
EMPOWER: Eliminating Medications Through Patient Ownership of End Results
EMPOWER-ED: Eliminating Medications Through Patient Ownership of End Results Electronic Delivery
PI: principal investigator
VHA: Veterans Health Administration
